# Single-cell T-cell landscape in atherosclerosis: implications for targeted therapy and beyond

**DOI:** 10.1186/s12967-026-08334-4

**Published:** 2026-06-10

**Authors:** Renfei Luo, Shixing Liu, Xin Ouyang, Enge Zhou, Long Jiang

**Affiliations:** Department of Cardiovascular Medicine, The Second Affiliated Hospital of Nanchang University, Nanchang, Jiangxi China

**Keywords:** Atherosclerosis, Single-cell RNA sequencing, T cell

## Abstract

**Background:**

Atherosclerosis (AS) progression is profoundly influenced by dynamic T-cell heterogeneity and functional plasticity.

**Main body:**

This review consolidates the latest insights derived from single-cell transcriptomic technologies, which are transforming our understanding of the role of the immune system in plaque pathogenesis. Recent evidence revealed previously unrecognized T-cell subsets within murine and human atherosclerotic lesions, distinguished by unique transcriptional profiles and adaptability driven by their microenvironment. Spatial mapping techniques have revealed compartment-specific distributions of T cells across vascular layers, whereas temporal analyses have highlighted age-related changes in the balance between effector and regulatory functions. Notably, single-cell resolution delineated transitional states between cytotoxic and regulatory lineages, suggesting that local inflammatory signals play a crucial role in determining T-cell fate. Despite these advancements, challenges remain in fully understanding T-cell lineage commitment, plasticity, and interactions with vascular niches. The integration of emerging multiomics approaches and spatial transcriptomics holds promise for addressing these challenges, providing a roadmap for novel therapeutic strategies. This includes not only the development of targeted immunotherapies but also the identification of key molecular targets for drug intervention and precision diagnostics, ultimately broadening the horizon for clinical applications in atherosclerosis.

**Conclusions:**

This review synthesizes single-cell RNA sequencing-driven discoveries in AS immunology, highlighting the heterogeneity and plasticity of plaque T-cell subsets. These insights lay the groundwork for developing targeted immunotherapies and identifying novel molecular targets and diagnostic biomarkers, ultimately advancing precision medicine for AS patients.

**Graphical Abstract:**

**Figure:**
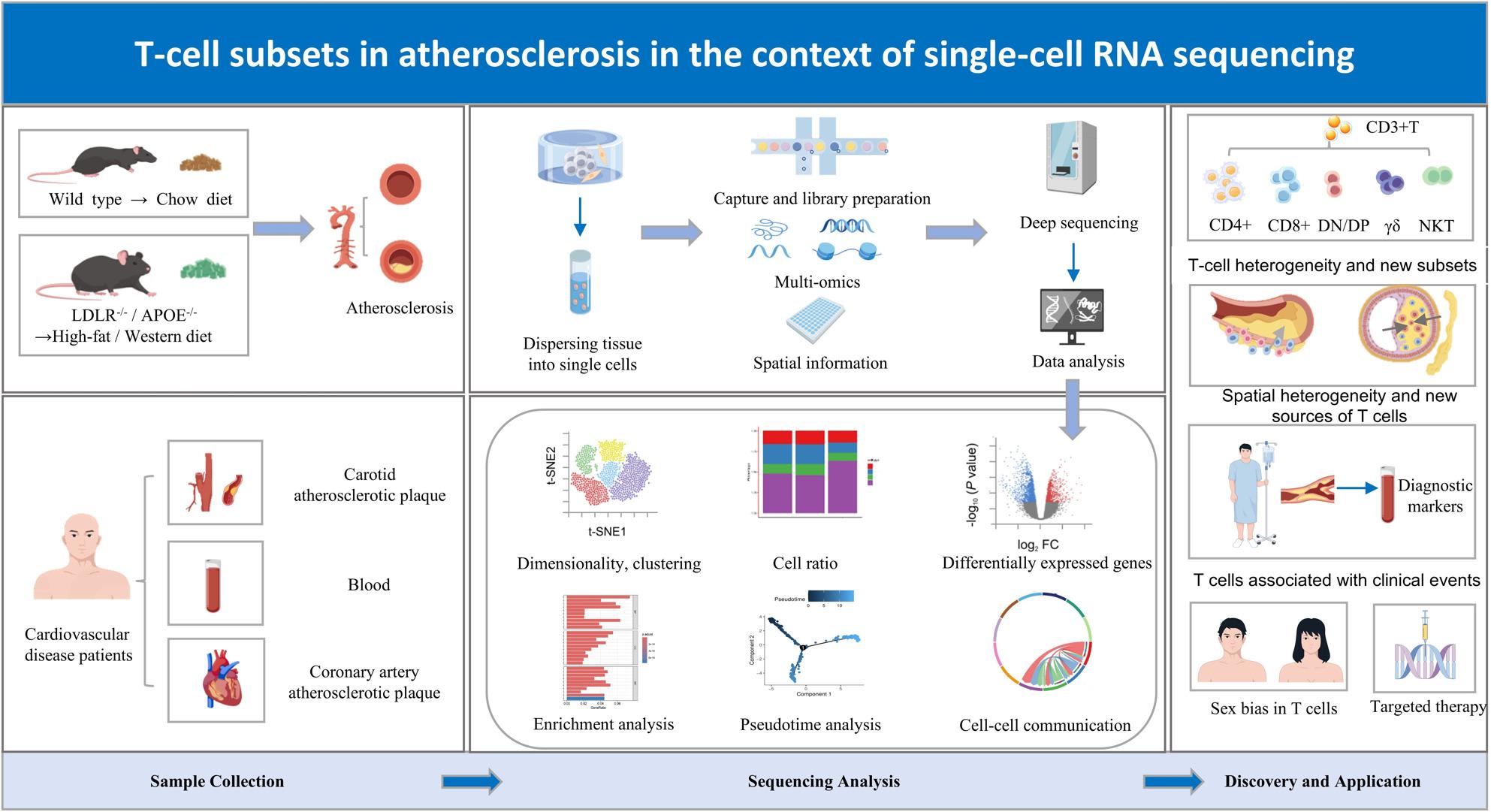
T-cell subsets in atherosclerosis in the context of single-cell RNA sequencing (scRNA-seq). Based on scRNA-seq technology and integrating mouse aortic and human plaque and peripheral blood immunoprofiles, the heterogeneity and functional characteristics of T-cell subsets in atherosclerosis have been revealed by using strategies such as differential gene expression, trajectory analysis, and cellular interactions, and have partially explained the issues of T-cell origins, gender differences, and mechanisms of clinical associations. The screening of key genes or cell populations provides precise intervention targets for targeted immunotherapy.

## Introduction

Atherosclerosis (AS), which originates from the injury of vascular endothelial cells, is a chronic inflammatory disease in which oxidized low-density lipoprotein is deposited under the endothelium of the great arteries, followed by the activation of immune cells [1]. The inflammatory microenvironment is composed of various immune cells, including macrophages, T cells, B cells, and natural killer cells, and they are involved in the dynamic process of AS from the initial formation of plaques to their rupture, and ultimately to ischemia [2]. In previous studies, macrophages have received increasing attention and have been shown to play a dual role in promoting plaque formation and plaque regression in AS [3]. In the continuous exploration of T lymphocyte populations, different types of CD4^+^T and CD8^+^T cell subsets have been shown to mediate specific immune responses with similar dual effects in the progression of AS [4]. Th1 cells, as the most prominent CD4^+^T cell group in plaques, have been shown to promote the inflammatory response and AS progression, but the functions of some CD4^+^T cells are controversial [5]. During the early stages of AS, CD8^+^T cells promote the formation of necrotic cores and instability of plaques, but provide local protective effects in the late stages of the disease [6]. Regulatory T cells (Tregs) with CD4 and CD8 phenotypes, which play a protective role in AS through immunosuppression, can also promote AS [4]. The heterogeneity of T-cell subsets highlights their functional complexity in AS, and further analysis of immune cell components in plaques is beneficial for the development of new targeted treatment strategies.

Early studies on immune cells in AS lesions mainly relied on immunohistochemistry [7], which characterized myeloid cells, T cells, and smooth muscle cells through a limited number of markers. Subsequently, flow cytometry with up to 18 parameter numbers was introduced, allowing for the staining of transcription factors, which led to a more in-depth analysis of infiltrating immune cells [8]. However, the insufficient number of markers and overlapping expression of detection signals limited further understanding of cell subpopulation heterogeneity. The new high parameter technologies have overcome these limitations. Cytometry by Time-Of-Flight (CyTOF), which is based on flow cytometry, uses heavy metal isotopes instead of fluorescent groups to label antibodies, expanding the number of antibody targets to 40 to 50, and has been widely used in the study of immune cells [9]. However, this method cannot provide single-cell transcriptional information. On the other hand, single-cell RNA sequencing (scRNA-seq) technology has achieved high sensitivity and large-scale transcriptome analysis, and has played an important role in the study of infiltrating T-cell subsets in AS [10].

Compared to conventional RNA sequencing, scRNA-seq resolves transcriptomes at the individual-cell level, circumventing population-averaged signals that obscure cellular heterogeneity and enabling systematic dissection of cell-type identity, state transitions and functional diversity. Since its inception in 2009, the technology has undergone continuous refinement in single-cell capture, isolation, library preparation, sequencing and bioinformatics, yielding substantial gains in scale, accuracy and sensitivity [11]. The throughput of scRNA-seq has increased dramatically from dozens of cells per experiment to hundreds of thousands in a single run. Micro-well arrays and droplet-based microfluidics have further automated high-throughput scRNA-seq while substantially reducing per-cell costs. Currently available scRNA-seq platforms include Fluidigm C1, SMART-seq2, BD Rhapsody (microwell-based), and 10x Genomics (droplet-based) [12]. Different platforms exhibit varying sensitivities in transcript recovery and gene coverage. The former two platforms excel in full-length transcript capture and ultra-high sensitivity, whereas the latter two employ molecular barcoding to markedly reduce experimental cost and increase cell throughput at the expense of some sensitivity, thereby facilitating the detection of low-abundance transcripts and the discovery of rare cell subpopulations. The breakdown of technical barriers is overcoming the limitations of conventional scRNA-seq, expanding the scope of cellular information capture toward a multidimensional multi-omics panorama [12]. For example, cellular indexing of transcriptomes and epitopes by sequencing (CITE-seq) enables simultaneous transcriptomic and proteomic profiling at single-cell resolution, offering a comprehensive perspective on cellular heterogeneity (Table 1) [13]. In the field of cardiovascular research, there has been a rapid growth in publications utilizing scRNA-seq, with particularly notable contributions in delineating immune cells within atherosclerotic plaques. From early exploration of T-cell subset heterogeneity to in-depth analysis combined with multiomics, scRNA-seq has revealed the complexity of the T-cell immune response in AS [14].

**Table 1:** High-throughput technology applied in conjunction with ScRNA-seq

Technology	Full Name	Molecular Layer	Principle	Cell Isolation	DNA/RNA/protein Separation	Key Applications in AS Research	Advantages	Limitations
CyTOF	Cytometry by Time-Of-Flight	Proteome	Detects protein expression using metal-tagged antibodies and mass spectrometry	Flow Cytometry	Antibody-Metal Conjugate Binding (Targets Proteins)	High-dimensional immunophenotyping of T cells in blood and tissue	No spectral overlap, deep profiling	No transcriptomic data, typically requires fresh samples, expensive, cells are destroyed
scRNA-seq	Single-Cell RNA Sequencing	Transcriptome	Captures transcriptomes of individual cells via barcoding and high-throughput sequencing	10x Genomics Chromium, BD Rhapsody, Flow Cytometry	Bead-Based Capture (Targets mRNA poly-A tails)	Identifying T-cell subsets, heterogeneity, activation states, plasticity, and lineage tracing	Whole transcriptome; unbiased; rare-cell detection; trajectory inference	No proteome; 3′-bias; dissociation artifacts; high cost
scATAC-seq	Single-Cell Assay for Transposase-Accessible Chromatin using sequencing	Epigenome	Tn5 transposase inserts adapters into open chromatin regions	10x Genomics Chromium (Fixed Nuclei)	Tagmentation (Targets Open Chromatin; Tn5 Transposase)	Studying epigenetic regulation and chromatin accessibility in plaque T-cell differentiation	Reveals transcriptional regulatory mechanisms, complementary to transcriptomics	Lower throughput, more complex data interpretation
CITE-seq	Cellular Indexing of Transcriptomes and Epitopes by Sequencing	Transcriptome, Proteome	Simultaneously measures mRNA and surface protein expression in single cells	10x Genomics Chromium	Bead-Based Capture (mRNA) + Antibody-Derived Tag Capture (Protein)	Integrated transcriptomic and proteomic profiling of T cells in plaques and blood	Multi-modal; protein-RNA co-detection; scalable	Surface proteins only; antibody panel design critical; cost
scTCR-seq	Single-Cell T Cell Receptor Sequencing	Genome	Targeted amplification of TCRα/β (or γ/δ) transcripts	10x Genomics Chromium, Flow Cytometry	Targeted PCR Amplification (Targets TCR V(D)J regions)	Identifying antigen-specific T-cell clones, autoimmunity, and viral cross-reactivity in plaques	Clonality tracking; antigen specificity (with tetramers); pairing accuracy	Requires RNA-quality cells; does not identify antigen without peptide-MHC data
Stereo-seq	Spatio-Temporal Enhanced Resolution Omics-sequencing	Transcriptome	Captures genome-wide transcriptomic data at subcellular resolution using DNA nanoball-based patterned arrays	No cell isolation required (Tissue sections)	In Situ Capture (High-density spatial barcodes on DNA nanoball arrays)	High-precision mapping of T-cell distribution in plaques, adventitia, ATLOs; revealing spatial context of cellular communication	Subcellular resolution, large field-of-view, provides both cell type and spatial location	TB-scale data; high compute burden; antibodies not compatible

DNA, Deoxyribonucleic Acid; MHC, major histocompatibility complex; PCR, polymerase chain reaction; RNA, Ribonucleic Acid; TB, terabyte; ATLO, artery tertiary lymphoid organ

In this work, we explore several key issues from the perspective of scRNA-seq of T cells in the setting of AS: 1) the heterogeneity and functional characteristics of T-cell subsets in AS in different species and different tissues; 2) how scRNA-seq provides new insights into the immune mechanism research and targeted therapy of AS; and 3) the current challenges and future directions of single-cell sequencing in translational studies of AS.

## T-cell subsets in mouse AS models

Immune regulation in AS has been extensively studied, but the diversity of T-cell subsets and the role of specific T-cell subsets in disease progression are still poorly understood. The origin, phenotypic transformation, and role of T cells in AS were explored through scRNA-seq studies of the aortas of low-density lipoprotein receptor (LDLR)^-/-^ and apolipoprotein E (ApoE)^-/-^ mice fed different diets and at different time points.

### Heterogeneity of mouse aortic T-cell subsets

ScRNA-seq studies have shown that aortic T-cell heterogeneity varies among different AS mouse models, with differences in T-cell frequency, type, and subset proportions (Table 2). Compared with control mice, high-fat diet (HFD)-fed mice presented more diverse T-cell clusters (11 vs. 1 in healthy mice) [15] and lower T-cell frequencies. These differences are influenced by diet and feeding duration, which may be influenced by an increased proportion of myeloid cells [15]. In the study by Cochain et al. [16], there was a significant difference (28.3% vs. 40.3%) in the frequency of T cells in the aortas of HFD-fed LDLR^-/-^mice (11 and 20 weeks), indicating that the contribution of T cell immunity increases as AS progresses. However, in western diet (WD)-fed ApoE^-/-^ mice (12 weeks), this proportion reached 38.2%, indicating that ApoE^-/-^ mice are more capable of inducing AS and activating T-cell immunity than are LDLR^-/-^ mice [16, 23]. However, attention should be paid to the transcriptional differences introduced by the different mouse models. For example, CD4^+^T cells from HFD-fed LDLR^-/-^ mice are enriched for stronger IL-18, IL-17, and pro-inflammatory signaling and are more immunologically characterized compared to other genotype/diet combinations [24]. Healthy mice predominantly have memory T cells (Tm), whereas atherosclerotic aortas are rich in proinflammatory CD4^+^ and CD8^+^T cells [15, 16]. However, the reported T-cell subset differences may be due to cell annotation bias, as reannotation of public datasets revealed discrepancies in the original findings [22]. These findings indicate that under hyperlipidemic conditions, the aortic immune environment and T-cell composition change, potentially revealing intrinsic regulatory mechanisms linked to disease development.

**Table 2:** T-cell subsets in the aorta of atherosclerotic mice

Author	Research type	Platforms	Separation strategy	Dataset	Mouse type	Diet	Sample size	Tissue and cell type	Total number of cells	Sequencing methods	Number of clusters of immune cells	Number of T-cell clusters	T cells ratio(CD8/T)	T-cell species
Winkels et al. [15]	Original research	10x Genomics	FACS	/	ApoE^-/-^	8-weeks, healthy	10	Aortic CD45^+^ cells	555	scRNA-seq	5	1	/	Memory T cells
					ApoE^-/-^	8-week,WD/CD-fed for 12 weeks	10/10	Aortic CD45^+^ cells	2,077/909	scRNA-seq	11	5	49%	Memory T cells,Th17,Th2, CD8^+^T,CD4/CD8 T
Cochain et al. [16]	Original research	10x Genomics	FACS	GSE97310	LDLR^-/-^	HFD/CD-fed for 11 weeks	8/9	Aortic CD45^+^ cells	956/401	scRNA-seq	12	4	28.30 (69.2%)	CXCR6^+^T,CD8^+^T cells,Mixed Tcells
					LDLR^-/-^	HFD-fed for 20 weeks	7	Aortic CD45^+^ cells	1,219	scRNA-seq	9	3	25.30 (66.4%)	CXCR6^+^T,CD8^+^T cells,Mixed T/B cells
					ApoE^-/-^	WD-fed for 12 weeks	10	Aortic CD45^+^ cells	/	scRNA-seq	13	5	38.20 (34.6%)	T cells,CXCR6^+^T,CD8^+^T cells
Smit et al. [17]	Original research	10x Genomics	FACS	Inclusion in dataset GSM2882368	LDLR^-/-^	WD-fed for 5 months, CD-fed for 22 months	29/12	Aortic CD45^+^ cells	5,286/6,430	scRNA-seq	15	10	65%,53%,42%	CD4^+^CD8^+^DP T,CD4^+^/CD8^+^ SP T,Tox^hi^T,Prolif.DP T,Gzmk^+^ CD8^+^T,Prolif.CD8^+^T,Il17a^+^γδ T,Treg,NKT,γδ T
Gu et al. [18]	Original research	10x Genomics	FACS	/	ApoE^-/-^, wild-type	WD-fed for 12 weeks	20/20	Aortic adventitial cells	3,153/2,271	scRNA-seq	15	3	20%	Mixed T,Th17,ILC2
He et al. [19]	Original research	10x Genomics	10x Genomics Chromium	/	C57Bl/6J mice	HFD-fed for 29 weeks	2	Aortic cells（Ful,Ascending,Arch,Thoradcic,Abdominal）	34,192	scRNA-seq	23	4	4.2%	Naïve T/Treg,Memory T,Th2,Th1
Sharma et al. [20]	Original research	10x Genomics	FACS	GSE141038	AAV-mediated PCSK9 transport constructs LDLR^-/-^ C57BL6/J mice	WD-fed for 20 weeks,+ CD-fed for 3 weeks (apoB-ASO+IgG/apoB-ASO+anti-CD25)	4/4/4	Aortic CD45^+^ cells	1,012/1,782/2,357	scRNA-seq	17	5	/	CD4^+^T,CD8^+^T,Mixed T,Treg,γδ T
Wolf et al. [21]	Original research	SMART-Seq,10×Genomics	FACS	GSE149070	ApoE^-/-^	1.8-week-old (apoB^+^/apoB^-^), WD/CD-fed for 12 weeks;	731/1012,295/129	Lymph node and aortic CD4^+^T cells	10/10;10/3	scRNA-seq	13/4	13/4	The proportions of “clonal” T cells that responded to lipoprotein B were 0.7% and 0.2%, respectively (CAD and control groups)	Treg/Th17/Th1/Tfh (ApoB^+^/ ApoB^-^)
Zernecke et al. [22]	Meta-analysis	/	/	Four original studies, including those by Cochain et al. [16] and Winkels et al. [15]	ApoE^-/-^, LDLR^-/-^	9 scRNA-seq datasets	/	Aortic cells		scRNA-seq	17	7	/	CD4^+^CD8^+^T,CD8^+^T,Il17^+^Cxcr6^+^T,Naïve T,Treg

apoB-ASO, apolipoprotein B-antisense oligonucleotide; ApoE, apolipoprotein E; CAD, coronary artery disease; CD, chow diet; DP, double positive; FACS, fluorescence activated cell sorting; HFD, high fat diet; ILC2, type 2 innate lymphoid cells; LDLR, low density lipoprotein receptor; NKT, natural killer T cell; scRNA-seq, single-cell RNA sequencing; Tfh, follicular helper T cell; Th, helper T cell; Tregs, regulatory T cells; WD, western diet

### Aortic T-cell subsets associated with aging

**Fig. 1:**
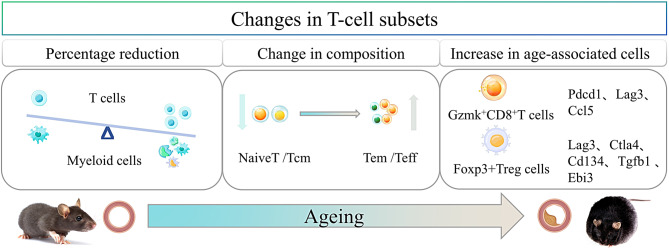
T-cell alterations in the aorta of aging mice. Aging drives a medullary shift, wherein the absolute number of T cells rises but their proportion within the total cell population declines as myeloid cells increase. Young aortas are dominated by naive T cells or central memory T cells (Tcm), while aged aortas have more effector memory T cells (Tem) or effector T cells (Teff). During aging, Gzmk^+^CD8^+^T cells and regulatory T cells (Tregs) increase significantly

Aging, as a driving factor of AS, affects immune changes in AS [25]. However, how aging affects the T-cell transcriptome and drives plaque progression remains unclear. The scRNA-seq of aged AS mice revealed that aging affects T-cell frequency and drives phenotypic switching. Under the same chow diet, the proportion of aortic T cells (42% <53%) in old LDLR^-/-^ mice (22 months) was lower than that in young LDLR^-/-^ mice (5 months) [17]. T cells in the aorta of young mice are mainly naïve and proliferating T cells, whereas they are dominated by activated effector memory T cells (Tem) and aging-related T cells in old AS mice (Fig. 1) [17]. In addition, the aortas of aged mice presented increased numbers of CD4^+^CD8^+^ double positive T (DPT) cells, CD4^+^T cells, and CD8^+^T cells. Among these, aged DPT cells exhibited gene expression profiles similar to those observed in early developmental stages, such as high expression of Rorc, Rag1, and Ccr9. Some DPT cells demonstrated high proliferative activity and cytotoxicity, with high expression of cell cycle-related genes (MKI67 and TOP2A) as well as Gzma. Aged CD8^+^T cells, particularly Gzmk^+^CD8^+^T cells, presented both effector (Gzmk, Ccl5, and Nkg7) and exhausted (Pdcd1 and Lag3) phenotypes. Flow cytometry data confirmed that the number of Gzmk^+^CD8^+^T cells increased approximately four-fold in the aortas of aged mice. CD4^+^T cells are predominantly Tregs, which highly express Ctla4, Lag3, and Tnfrsf4, and have increased expression of inhibitory cytokines such as Tgfb1 and Ebi3 in aged mice. Despite the increased number of Tregs with immune-suppressive functions, the overall CD4^+^T cells still exhibit a proinflammatory tendency (e.g., an increased proportion of IFN-γ^+^ cells), which may be related to the progression of atherosclerosis [17]. A discussion of senescent Gzmk^+^CD8^+^T cells was emphasized in a study by Tyrrell et al. [26]. The authors performed scRNA-seq on T cells enriched in plaques of young (5–month–old) and old (21–month–old) PCSK9 adeno-associated virus-treated wild-type mice, and the results revealed that the proportion of naïve CD8^+^T cells in both young and old plaques was very small(<10%) [26]. Old plaques have fewer CD8^+^ central memory T cells (Tcm) but more Gzmk^+^CD8^+^Tem expressing exhaustion and cytotoxicity markers, and trajectory analysis has revealed a developmental path from naïve CD8^+^T cells to exhausted Gzmk^+^CD8^+^Tem [26]. These results suggest that naïve T cells may migrate into plaques, then differentiate into memory T cells, and differentiate into a subset of exhausted CD8^+^Tm in the senescent state and accumulate in plaques. High expression of migration-related genes (e.g., CCR5, CXCR6, and CXCR3) by CD8^+^T cells in senescent aortas may promote T-cell migration toward atherosclerotic plaques [26]. Transplantation of aged CD8^+^T cells into young CD8^-/-^mice increased plaque formation, highlighting the role of senescent CD8^+^T cells’ role in AS progression [26]. In previous studies, CD8^+^T-cell depletion modulated AS by decreasing plaque macrophage accumulation and the number of circulating monocytes, reflecting the complex mechanisms by which senescent CD8^+^T cells influence AS [27]. In addition, plaques contain γδ T cells, which account for 30–45% of all T cells, and these cells enhance pathways associated with T-cell adhesion, actin regulation, and T-cell activation with age [26]. These results suggest that aging promotes changes in various T-cell subsets, thereby affecting the onset and progression of AS. However, further studies are needed to elucidate the mechanisms by which aging affects different T-cell subsets in AS.

### Spatial differences in mouse aortic T-cell subsets

**Fig. 2:**
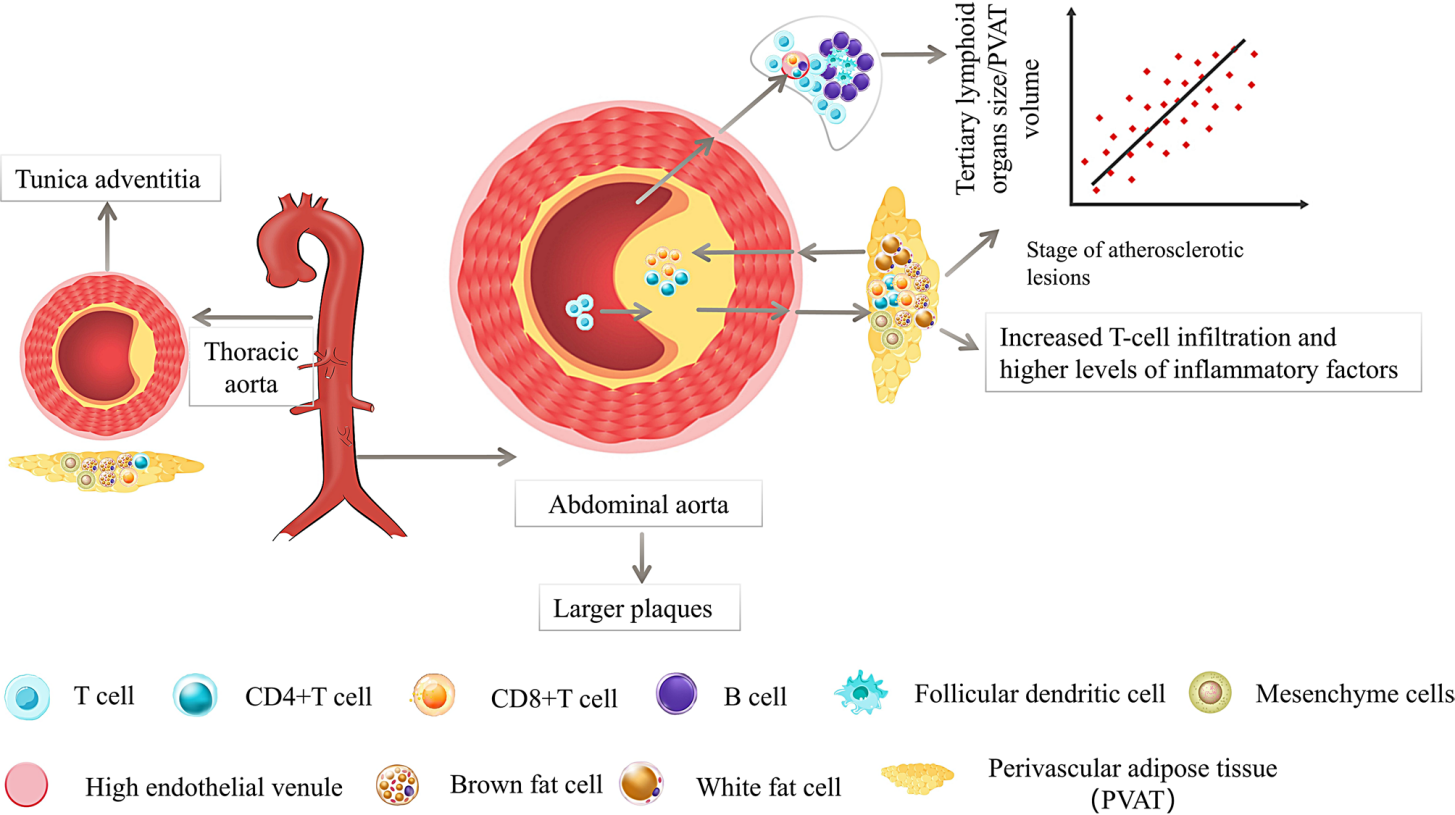
Spatial heterogeneity of aortic T cells. The aortic tunica is enriched with T-cell populations with characteristics similar to those of plaque T cells, and their aggregation correlates with the formation of artery tertiary lymphoid organs (ATLOs) in the tunica, and the size of the ATLOs is positively correlated with the degree of atherosclerosis. Bidirectional interaction of adjacent perivascular adipose tissue (PVAT) with the tunica influences the immune environment. The abdominal aorta presented stronger T-cell infiltration, inflammatory factor expression, and lesion susceptibility than the thoracic aorta

ScRNA-seq mapped the immune cell landscape of the aortic adventitia and partially explained the source of T cells at the lesion site. Previous studies have shown that lymphocytes recruited to the intima are thought to be exogenous and accompany the early stages of AS development [28]. There is a high enrichment of T-cell subsets in the aortic adventitia, which has been shown to be a major area of inflammation in the arterial wall [26]. The artery tertiary lymphoid organ (ATLO) in the adventitia is rich in T cells and B cells and is associated with late AS plaques (Fig. 2) [29, 30]. Furthermore, in the aortas of atherosclerotic mice (WD-fed, >60 weeks), the abundance of adventitial T cells is greater than that at the lesion site [31]. These findings suggest that T cells in plaques may originate from the adventitia. Gu et al. [18] provided a possible basis by performing scRNA-seq on the mouse aortic adventitia and found three similar T-cell clusters, constituting 21% and 20% of all cells, respectively, in ApoE^-/-^ and wild-type mice (both chow diets). CD8a-expressing T cells predominated, aligning with their abundance in lesion sites. T helper cell 17 (Th17) and Th2, which are also found in lesions, are present in the adventitia, suggesting a link between the adventitia and lesion T-cell subsets. The mechanism of T-cell recruitment to the adventitia is unclear but involves specific ligand-receptor networks. For example, mesenchymal cells expressing Sca-1 in the adventitia attract immune cells, including a subset of Tm, via CCL2 secretion, potentially influencing T-cell residence in adventitial inflammation [18]. In addition, studies have shown that there is a positive correlation between the volume of perivascular adipose tissue (PVAT) and the progression of AS, and immune cells that accumulate under oxidative stress may be transported to the adventitia, ultimately affecting the formation of AS [32]. The complex immunoregulatory relationships among the aortic lesion site, adventitia, ATLO and PVAT are currently unclear. Further research is expected to elucidate the intrinsic relationship and to clarify the homing problem of T-cell subsets in the aortic lesion site.

ScRNA-seq can elucidate the spatial distribution of T-cell subsets across various aortic segments. In healthy aortas, the naïve/Treg subset constitutes 47% of the T-cell population and is predominantly found in the aortic arch and abdominal aorta, possibly due to vessel wall damage from high axial and shear stress [19]. Under high-fat conditions, the proinflammatory Th17 and cytotoxic CD8^+^T cells remain enriched in the abdominal aorta. Studies have indicated that the abdominal aorta is more prone to AS than the thoracic aorta is [33]. Furthermore, T-cell infiltration and inflammatory factor expression are more pronounced in the PVAT of the abdominal aorta than in that of the thoracic aorta [34]. The specificity of T-cell immunity in the aorta and its relationship with differences in the PVAT warrant further investigation. Aortic scRNA-seq studies alone are insufficient to address aortic spatial heterogeneity. Spatial transcriptomic technologies can reveal differences in cellular distribution from the outer membrane to the plaque in this axial direction, information that is often missed in conventional single-cell sequencing [35]. In addition, multi-omics analyses integrating spatial transcriptomic data from multiple organizational regions are expected to provide deeper mechanistic insights into the problem.

### ScRNA-seq reveals plasticity of mouse aortic T-cell subsets

ScRNA-seq can be used to explore the phenotypic transformation of T-cell subsets during the progression of AS to reveal the immune regulatory mechanism of AS development. Recent studies confirmed that the differentiation of TH cell lineages is flexible and that these cells can differentiate into other or mixed phenotypes or into other TH cell subsets [5, 20, 21]. Tregs in progressive plaques highly express activation-related genes (Ctla4 and Itgb1). However, Tregs in regressing plaques highly express factors related to their differentiation or maintenance (Ly6a, Mif, and Igals9) and have increased expression of metabolically active genes [20]. In another scRNA-seq study, attenuated Treg phenotypic expression in the aortas of WD-fed ApoE^-/-^ mice compared with the healthy control mice was accompanied by a selective enhancement of proinflammatory Th1/Th17 phenotypes, confirming the possible conversion of Tregs to proinflammatory CD4^+^T cells during AS progression [21]. However, the transition of Tregs to Th17 was not confirmed in the study by Depuydt et al. [36], as no overlapping phenotypes were detected between the two groups. It remains uncertain whether this finding was due to an insufficient number of detected T cell receptors (TCRs). Furthermore, Tem in plaques also undergo phenotypic conversion during the progression of AS. Fan et al. [37] identified LMNA^+^Tem, PDCD1^+^ Tem, and NKG7+ effector memory T cells re-expressing CD45RA (NKG7+ Temra) among both CD4^+^T and CD8^+^T cells via scRNA-seq of plaque immune cells. The subsequent paired scRNA-seq results revealed significant clonal expansion and local differentiation among the three T-cell subsets. By employing RNA velocity [38], CytoTRACE [39], and Monocle3 [40] for trajectory analysis of differentiation, they inferred a differentiation process from NKG7^+^Temra cells to PDCD1^+^Tem and subsequently to LMNA^+^Tem, accompanied by a gradual downregulation of cytotoxic genes (PRF1 and NKG7) and upregulation of T-cell activation-related genes (CD44, NFKBIA, FOS and LMNA). This result shows that Tem within the plaque progressively lose their degree of cytotoxicity and transition toward a persistently activated state. Research relying on scRNA-seq could help reveal the phenotypic changes in each T-cell subset during the progression of AS and further analyze the immune regulation of T-cell subsets at different stages of AS.

### Identification of novel T-cell subsets in the mouse aorta

**Fig. 3:**
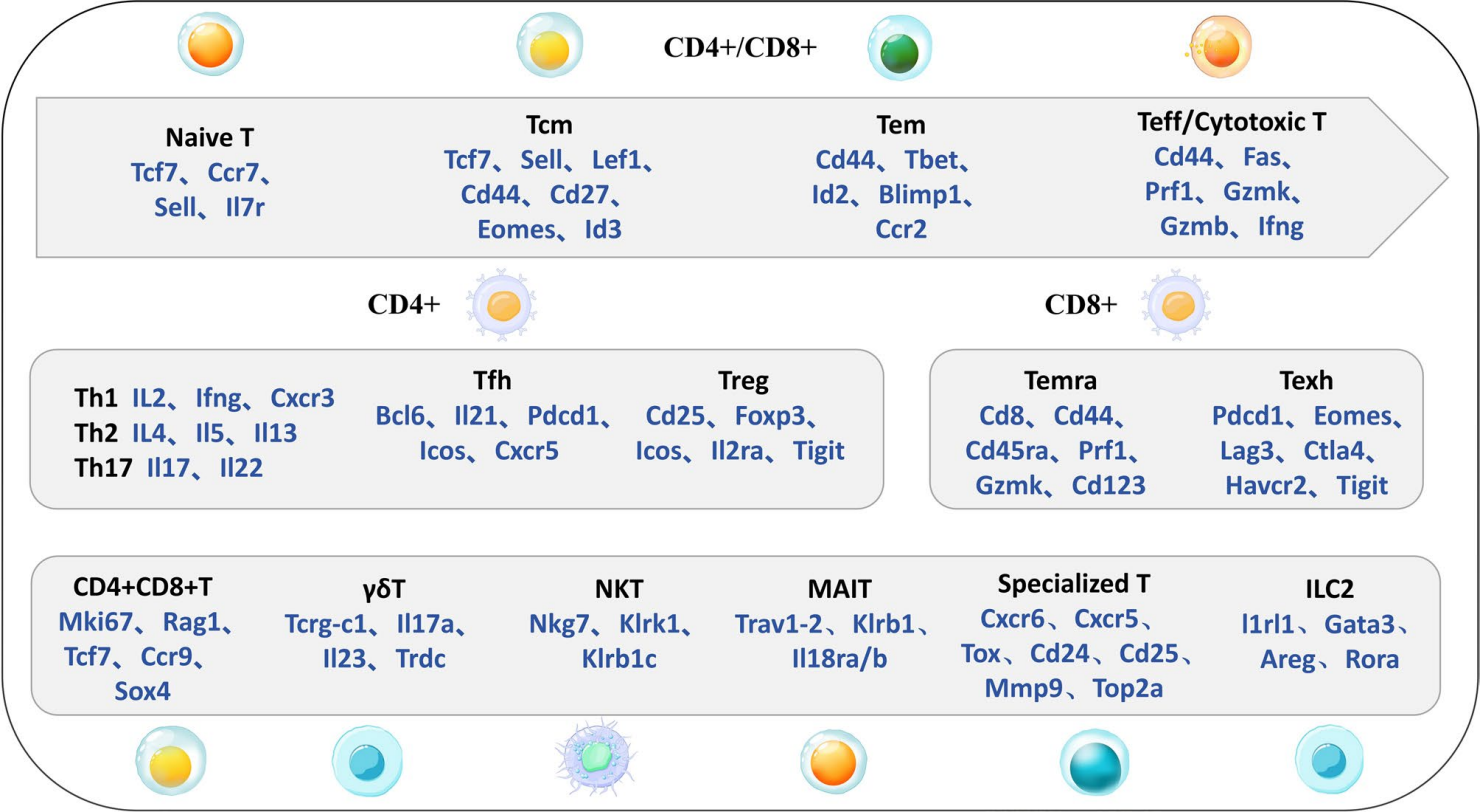
Summary of detectable T-cell subsets in atherosclerotic aorta. The identification and characterization of T-cell subsets in aortic tissues based on expression profiling of specific cell marker genes and differential gene expression analysis allows annotation of, but is not limited to, the above T-cell subsets. ILC2, type 2 innate lymphoid cell; MAIT, mucosal-associated invariant T cell; NKT, natural killer T cell; Tcm, central memory T cell; Teff, effector T cell; Tem, effector memory T cell; Temra, effector memory T cell re-expressing CD45RA; Texh, exhausted T cell; Tfh, follicular helper T cell; Th, helper T cell; Treg, regulatory T cell

ScRNA-seq allows a comprehensive understanding of previously identified or undetected T-cell subsets to provide complete cellular profiling information. A cluster of immature DPT cells that specifically highly express chemokines (Ccr9) and genes that regulate lymphocyte proliferation and development (Tcf7, Rag1 and Sox4) was identified in the aorta, indicating that this type of cell cluster may have the potential to differentiate into other cells (Fig. 3) [22]. DPT cells are thought to originate in the thymus and migrate to the aortic arch in a manner independent of SAP1—a G protein-coupled receptor that mediates lymphocyte egress from lymph nodes and thymus into circulation—where they may further differentiate into CD4-biased single-positive T cells [41]. Smit et al. [17] first defined a population of depleted CD8^+^T cells in the aortas of aged LDLR^-/-^ mice that expressed both depleted genes (Eomes, Pdcd1, and Lag3) and effector molecules (Gzmk, Nkg7, and Gzmb), suggesting a progressive loss of T-cell effector function in the senescent state. Tyrrell et al. used TCR-seq for the aorta and reported that this type of CD8^+^T cells undergoes significant clonal expansion during aging [26]. In addition, type 2 innate lymphoid-like cells (ILC2) do not express CD3 and highly express a cellular marker (Il1rl1) and a transcription factor (Gata3) [18, 22]. ILC2s in PVAT have been previously shown to promote AS through the secretion of type 2 cytokines (IL-3 and IL-5) [42]. ScRNA-seq revealed that the ILC2 cluster in the aortic epithelium upregulated the expression of Il1b, Fosl2, and genes related to oxidative phosphorylation, suggesting that this cell cluster may play a regulatory role in the early stages of AS [18]. Finally, a small number of γδ T cells, which express Il23 and Il17a, have been identified in the aorta [17, 20, 43]. IL-23 R γδ T cells are located predominantly in the aortic root and can promote early AS formation [43], and bone marrow-derived γδ T cells can promote AS and plaque instability [44]. With increasing research, new cell types will continue to be discovered, and T-cell functions will be deeply analyzed from complex cell subsets, providing strong support for further elucidation of the AS mechanism.

## T-cell populations in human atherosclerotic plaques

ScRNA-seq studies of T-cell subsets in human AS lesions and mouse AS models have yielded consistent results. A comprehensive analysis of public sequencing datasets of human carotid atherosclerosis samples revealed that plaque T-cell subsets similarly represent one of the largest populations of leukocytes [45]. However, plaque phenotypes derived from mouse atherosclerosis models do not fully represent those observed in human samples [46]. The scRNA-seq studies in human samples have demonstrated methodological diversity. A combined strategy of multiple histologic approaches provides insight into alterations in T-cell subsets in the dysregulated plaque microenvironment.

### Characteristics of T cells in human atherosclerosis

Similar to the proportion of T cells in mouse samples (25%–60%), T cells were predominant in single-cell sequencing analyses of human plaques (31%–52%), even reaching 65% in CyTOF analyses [47]. However, in the immunohistochemical analysis of carotid atherosclerotic plaques, T cells were presented at low percentages (5%–25%) [7]. Possible reasons for this discrepancy in results are the loss of macrophages during single-cell suspension preparation and the varying degrees of plaque development among the samples studied. Notably, T cells may constitute a more predominant leukocyte population in human atherosclerotic plaques compared to murine models. In a cross-species scRNA-seq investigation integrating data from eight human carotid endarterectomy specimens and seven murine aortic scRNA-seq datasets, Horstmann et al. [24] observed that T cells accounted for 65%–67.8% of leukocytes in human plaques, whereas atherosclerotic mouse aortas contained only 36.6% ± 9.4% T cells on average—a discrepancy potentially attributable to the dilutional effect of the arterial adventitia on plaque-derived cells in mice. The proportion of T cells was greater in stable plaques (57.2% vs. 36.7%), indicating that plaque type also affects T-cell abundance (Table 3) [54]. In addition, the proportion of CD8^+^T cells in the plaques was similar to that of CD4^+^T cells, but the change in the abundance of CD8^+^T cells was greater. In mouse plaques, CD8^+^ effector T cells (Teff) are more significantly expanded than CD4^+^Teff are (36% vs. 9.7%) [55]. Compared with carotid plaques, femoral plaques exhibit relatively anti-inflammatory features with a significantly lower proportion of toxic CD8^+^T cells (40.1% vs. 27.8%) [56]. Chowdhury et al. examined the expansion of T-cell subsets in coronary plaques by ScTCR-seq and reported that predominantly CD8^+^T cells were expanded in plaques [48]. However, ScTCR-seq of carotid atherosclerotic plaques by Depuydt et al. revealed the highest degree of plaque-specific clonal expansion in CD4^+^Teff [36]. These findings highlight that the roles of different T-cell subsets in plaque progression vary, likely due to differences in plaque location and stage. In terms of activation status, T cells in plaques are dominated by T cells of the effector phenotype, including Tem and Teff [47, 48]. The progressive absence of Tm cell subsets in plaques and the increase in effector T cells increase the risk of ischemic events [15]. Differences in study methodology, sample source, or disease stage can lead to differences in the immune milieu and the contribution of T-cell subsets, reflecting the complexity of immune cell regulation in plaques. More scRNA-seq studies are needed to capture the ability of different T-cell subsets to contribute to AS. The origin of T cells in plaques has not yet been elucidated. The application of spatial transcriptomics has provided deeper insights into the origin and regulation of immune cells within plaques. Through integrated analysis of Stereo-seq and scRNA-seq data, Lai et al. not only identified ATLOs and their specific transcriptional profiles in human atherosclerotic plaques, but also revealed migratory activity of B cells between ATLOs and PVAT [57]. Predictably, the origin and migration of T cells in plaques will be answered in similar studies.

**Table 3:** T-cell subsets within plaques and in the blood of patients with atherosclerosis

author	Research type	Platforms	Separation strategy	Dataset	Sample size	Tissue and cell type	Total number of cells	methods	Number of cell clusters	Number of T-cell clusters	T-cell ratio	T-cell annotations
Depuydt et al. [36]	Original research	10x Genomics	FACS	10.34894/DDYKLL (dataverse.nl)	3	Carotid atherosclerotic plaques,PBMC (CD45^+^ cells)	33,249	scRNA-seq,scTCR-seq	13	12	/	Tm,naïve T,Te,Treg,Texh,γδ-T,MAIT
Fernandez et al. [47]	Original research	10x Genomics	CD45^+^ magnetic bead enrichment	https://figshare.com/s/c00d88b1b25ef0c5c788	46(6 plaque samples for ScRNA-seq）	Carotid atherosclerotic plaques,PBMC (CD45^+^ cells）	7,169	CITE-seq,scRNA-seq,CyTOF	12（*n* = 15，plaques,CyTOF）	7	65%,41%(ScRNA-seq)	CD4^-^CD8^-^T,CD4^+^Tcm CD27^+^CD25^int^ T,CD4^+^Tem CD27^+^CD25^hi^T,CD4^+^CD8^+^T,CD4^+^Tem,CD8^+^Tem,CD8^+^Temra
Chowdhury et al. [48]	Original research	10x Genomics	FACS	GSE196943	12(plaques),3(paired blood samples),20(ScTCR-se)	Coronary plaques(CD45^+^ cells）	23,618	ScTCR-seq,scRNA-seq	5	7	Virus-specific clonal CD8^+^ T cells in plaques accounted for 0.99% of all clonal CD8^+^ T cells	Naïve T,Th17-like Tem,CTL CD8^+^Tem,regulatory Tem,NK-like T,T-myeloid like
Depuydt et al. [49]	Original research	10x Genomics	FACS	/	18	Carotid atherosclerotic plaques	3,282	scRNA-seq,scATAC-seq	14	8	52.4%	PRF1^+^Cytotoxic CD4^+^T,GZMK^+^ Cytotoxic CD4^+^T,IL7R^+^ Naïve CD4^+^T,FOXP3^+^Treg,LEF1^+^CD4^+^TCM,CD8^+^TEM,GZMB^+^ Cytotoxic CD8^+^T,LEF1^+^CD8^+^TCM
Tan et al. [50]	Original research	BD Rhapsody	Bead-based	/	20	Carotid atherosclerotic plaques and paired PBMC	372,070	scRNA-seq	19	17	32.87%,GZMK^+^MALAT1^+^CD8^+^Te 11.8%,GZMK^+^NR4A1^+^CD8^+^Te 15.5%,CDN1A^+^NR4A^+^CD4^+^Te 10.7%	GZMK^+^NR4A1^+^CD8^+^Te,GZMK^+^MALAT1^+^CD8^+^Te,CDN1A^+^NR4A^+^CD4^+^Te,CCR7^+^Naïve CD4^+^T,LEF1^+^ Naïve CD8^+^T,FOXP3^+^Treg,GZMA^+^CD4^+^Te,GZMH^+^CD4^+^Te,IFI44L^+^ CD4^+^Te,ANXA1^+^CD4^+^Tm,GZMK/GZMH^+^ CD8^+^Te,ZNF683^+^ CD8^+^Te,SLC4A10^+^ CD8^+^Te,IFI44L^+^ CD8^+^Te,TRDC^+^TRGC1^+^γδT,TOP2A^+^Proliferating T
Vallejo et al. [51]	Original research	BD Rhapsody	Bead-based	GSE205320	31	PBMC	41,611	scRNA-seq, CITE-Seq	50	27	/	IL2RA^+^Treg, CXCR3^+^Th1,CXCR5^+^Tfh，CD56^+^CD4^+^T,CD56RA^+^Emra^+^CD45^+^NKT,CD2^+^CD8^+^T
Saigusa et al. [52]	Original research	BD Rhapsody	Microwell array	GSE190570	61	PBMC (CD4^+^T cells)	162,454	scRNA-seq,CITE-Seq	16	16	25.13%	PDL1 Tem,Tcm,Th1,PD1^+^Tem,CD24^+^T,PDCD1^+^T,MMP9^+^T,Th2,CD127^-^Naïve T,Naïve T,Tfh,CD123^+^Temra,CCR2^+^Tem,Treg,Cytotoxic Tem
Xiong et al. [53]	Meta-analysis	/	/	GSE159677,GSE155512,GSE131778	3,3,8	Carotid atherosclerotic plaques	44,120	scRNA-seq	6	10	54.81%	SELL^+^CD4,CXCR5^+^CD4,FOXP3^+^CD4,GZMA^+^CD4,Unknown CD8,ZNF683^+^CD8,IFI44L^+^CD8,GZMK^+^CD8,GZMB^+^CD8,TOP2A^+^CD8

CITE-seq, cellular indexing of transcriptomes and epitopes by sequencing; CTL, cytotoxic T lymphocyte; CyTOF, cytometry by time-of-flight; FACS, fluorescence activated cell sorting; MAIT, mucosal-associated invariant T cell; PBMC, peripheral blood mononuclear cell; scATAC-seq, single-cell assay for transposase-accessible chromatin using sequencing; scRNA-seq, single-cell RNA sequencing; scTCR-seq, single-cell T cell receptor sequencing; Tcm, central memory T cell; Te, effector T cell; Tem, effector memory T cell; Temra, effector memory T cell re-expressing CD45RA; Texh, exhausted T cell; Tfh, follicular helper T cell; Tm, memory T; Th, helper T cell; Treg, regulatory T cell

### T-cell subsets in human plaques

The application of scRNA-seq technology has provided new perspectives for in-depth investigations of the heterogeneity of T-cell subsets in carotid and coronary atherosclerotic plaques. Depuydt et al. [49] identified eight T-cell clusters in carotid intima-media plaque tissue, including toxic T cells expressing PRF1, GZMK, and GZMB; naïve T cells; and Tcm, Tem, and Treg. In another study, exhausted T cells expressing exhaustion genes (PDCD1, HAVCR2, and TOX), as well as a small number of γδ T cells and mucosa-associated invariant T (MAIT) cells, were identified in carotid plaques, and a large number of Teff expressing toxicity genes (GZMA, GZMK, and GZMB) were characterized (Table 4) [36]. MAIT cells, as innate-like T cells, exist mainly in human mucosal tissues and blood [58]. They are activated in a TCR-dependent or TCR-independent manner and perform rapid effector functions [58]. However, their role in the field of atherosclerosis is unclear. In addition, Chowdhury et al. [48] annotated Natural Killer T cells (NKT) expressing NKT receptors and T-myeloid-like cells expressing LYZ in coronary atherosclerotic plaques. NKT cells act as lipid-sensing cells that promote the progression of atherosclerosis [59]. Different annotation results can be obtained depending on the key highly expressed genes. Xiong et al. [53] annotated CD8^+^T cells specifically expressing ZNF683 (transcription), IFI44L (IFN-induced) and TOP2A (proliferation) in plaques. With the continuous development of single-cell sequencing studies and improvements in histological databases, T cells with different functions and phenotypes will be identified to better reveal the complexity and diversity of T-cell subsets in atherosclerosis [47].

**Table 4:** Diagnostic markers and potential therapeutic targets for atherosclerosis

Gene/Marker	Cell Type/Source	Functional Characteristics	Potential Drug/Application	Research Evidence	Ref.
PAM	Immune cells	Key DEG in unstable plaques;	Diagnostic marker for plaque instability	Bioinformatics analysis of human plaques	[45]
IGFBP6,CNN1/SLC3A2	CD8+T cells	Positively/Negatively correlated with CD8+T cells	Marker for plaque assessment,Diagnostic indicator for plaque status	miRNA-core gene network, Bioinformatics analysis	[45]
MDM2,KCNA5,ANO1	Immune cells	Potential therapeutic target for AS treatment	Multiple targeted drugs available	Gene-drug interaction mining	[45]
CXCR4, CCL4L2	CD8+T cells	Chemotactic genes expressed in ASYM plaques	Markers for plaque stability assessment	DEG analysis of human plaques	[47]
CD11c	Activated CD4+T cells	Dendritic cell maeker, Activated CD4+T cells in fragile plaques; secrete increased IFNG	Marker for plaque vulnerability	scRNA-seq of human plaques	[54]
IFI44L	CD8+T cells in plaques	Proinflammatory subset; upregulated IFN signaling; increases with AS progression	Target for inhibiting pro-AS T cells	scRNA-seq meta-analysis	[53]
ZNF683, IFI44L, TOP2A	CD8+T cells in plaques	transcription, IFN-induced, proliferation	Diagnostic and therapeutic targets	scRNA-seq annotation	[53]
GZMA,PRF1, GZMK, GZMB,XCL1	Toxic T cells in plaques	Cytotoxicity markers,NF-κB pathway regulation, proinflammatory	Target for controlling inflammation	scRNA-seq meta-analysis,Human CITE-seq (SYM plaques)	[36, 49, 53, 80]
GZMK,NR4A1,MALAT1,CDN1A	Te cells (plaques)	Plaque-specific T cell subset; promote AS	Target for inhibiting plaque progression	scRNA-seq of carotid plaques	[50]
HAVCR2, TOX	Exhausted T cells in plaques	Exhaustion markers; preferentially distributed in plaque cores	Monitor exhaustion status; PD-1 targeted therapy	scRNA-seq of human plaques	[36, 47, 50]
IL32	PBMC T cells	Inflammatory cytokine; increased in CAD patients	Biomarker for cardiovascular disease	scRNA-seq of CAD patient PBMC	[69]
JUMB, LCK	PBMC T cells	Promote Th17 development; upregulated in CAD, decreased after LLT	Biomarkers for treatment response	scRNA-seq of CAD patient PBMC	[69]
RUNX	PBMC T cells	Reduced in disease, increased during LLT treatment	Biomarker for treatment monitoring	scRNA-seq of CAD patient PBMC	[69]
PD-1/PDCD1	Exhausted T cells in plaques	Widespread use of PD-1 inhibitors may exacerbate AS; FcγR+anti-PD-1 mAb reduces plaque size	Dual role: caution in cancer patients; potential targeted therapy	Clinical studies in tumor patients with AS	[77]
MMP9/12	Immune cells	Matrix metalloproteinase; anti-AS effect through MMP9/12 targeting	Salvianolic acid B	Bioinformatics validation	[80]
TCF7	T cells (mouse aorta)	Proliferation-related gene; AKT pathway regulation	Under investigation	Mouse scRNA-seq	[81]
CCR9	T cells (mouse aorta)	T cell chemokine receptor; proliferation marker	Under investigation; T cell recruitment inhibition	Mouse aortic scRNA-seq	[81]

AS, atherosclerosis; ASYM, asymptomatic; CAD, coronary artery disease; CITE-seq, cellular indexing of transcriptomes and epitopes by sequencing; DEG, differentially expressed genes; LLT, lipid-lowing therapy; mAb, monoclonal antibody; PBMC, peripheral blood mononuclear cells; scRNA-seq, single-cell RNA sequencing; SYM, symptomatic;Th, helper T cell

### Characterization of T-cell subsets in plaques associated with clinical events in plaques

ScRNA-seq studies have explored immune dysregulation during plaque evolution to identify immune drivers that promote clinical cardiovascular events. Fernandez et al. reported that the CD4^+^Tem frequency was greater in symptomatic (SYM) plaques than in asymptomatic (ASYM) plaques (32% vs. 20%) [47]. Further results of differentially expressed genes (DEGs) and enrichment analyses revealed that CD4^+^T cells enhanced effector signaling pathways (IFNG, type I interferon, IL1, and IL6) in ASYM plaques, whereas they responded primarily to T-cell activation, migration, and differentiation programs in SYM plaques. In addition, CD8^+^T cells had specific chemotactic gene (CXCR4, CCL4L2) expression and enriched effector signaling pathways (type I interferon, IL-6, granzyme, and TGFβ) in ASYM plaques, whereas in SYM plaques, there was enrichment of the migration, differentiation, and IFN-γ pathways and unique enrichment of the senescence signaling pathway in SYM plaques, suggesting that the loss of T-cell function maintained by chronic plaque inflammation may also be caused by overactivation of T cells [47]. The differential signaling pathway enrichment profile of T-cell subsets in different plaques suggests that anti-inflammatory therapies may not be equally applicable at different stages of the disease [47]. In a study by Ge et al. [54], the frequency of CD4^+^Teff was found to be equivalent between stable and vulnerable plaques, whereas CD4^+^Tm was more prevalent within stable plaques. In addition, activated CD4^+^T cells expressing CD11c secrete increased levels of IFNG in fragile plaques [54]. Chowdhury et al. reported the highest proportion of activated cytotoxic CD8^+^ Tem in plaques, with increased numbers in complex plaques with more pronounced proinflammatory and lysogenic features [48]. Tan et al. [50] found that the proportion of Treg was significantly increased in SYM plaques and acquired the proinflammatory properties of some Th9 and Th17. Furthermore, in SYM plaques, CDKN1A^+^NR4A1^+^CD4^+^T cells were enriched in enhanced T-cell activation and proinflammatory pathways, and SLC4A10^+^CD8^+^T cells enhanced T-cell inflammation, depletion, chemoattraction, and differentiation-related pathways, which may have contributed to the cerebrovascular events. CD42 and CD2 are highly expressed signature genes in CDKN1A^+^NR4A1^+^CD4^+^T cells and SLC4A10^+^CD8^+^T cells, respectively. The expression of these genes was reduced in SYM plaques, and their expression levels were negatively correlated with the risk of cerebrovascular events, further confirming the proatherogenic role of these two cell populations. The positive response of T cells during plaque evolution may contribute to the generation of necrotic cores and the attenuation of fibrous adventitia leading to plaque rupture and ultimately facilitating clinical events. The full exploitation of T-cell transcriptional profiling information can help to clarify the intrinsic link between various T-cell subset subgroups and the occurrence of clinical events.

### Exploration of T-cell immune mechanisms in AS plaques by co-sequencing

Owing to the use of the scRNA-seq strategy, the transcriptional profiling information of different T-cell subsets in plaques has been sufficient. However, functional T-cell transcriptional regulatory mechanisms and clonal expansion programs have not been fully characterized. By determining the staining accessibility of T-cell populations through a combined transposase-accessible chromatin using sequencing (scATAC-seq) strategy, specific transcriptional regulatory mechanisms of T-cell subsets were explored. Depuydt et al. [49] reported that the chromatin of Nkg7 in T cells was open and enriched for transcription factor motifs (ETS1). The presence of chromatin opening of IFNG and TNF in Th1-like CD4^+^T cells may be related to the induction of a Th1-like cytotoxic phenotype by chromatin opening of IL12 in myeloid clusters. Specifically, the RUNX3, STAT3, and BATF-JUN motifs are enriched in CD4^+^T cells and are essential for the induction of cytotoxic gene expression and maintenance of cellular effector function [60, 61]. The chromatin opening of the GZMB, GZMH, and IL2 loci in CD4^+^T and CD8^+^T cells confirms the effector function of T cells in plaques [49]. Second, ScTCR-seq was able to monitor specific TCR clones in plaques [36]. The percentage of clonal expansion of T cells within plaques reached 29% and this plaque-specific expansion program suggests that the expansion program is induced by restriction antigens [36]. Clonally expanded T cells expressed T-cell activation markers (CD69, FOS, and FOSB), indicating that the TCR responded to a recent specific antigenic stimulus [36]. In the ScTCR-seq study by Chowdhury et al. [48], there was cross-reactivity between viral epitopes and self-epitopes of plaque T cells, and the resulting autoimmunity may explain the occurrence of cardiovascular diseases in the absence of active infection [48]. A comparison of the ScTCR-seq dataset of AS patients with that of psoriatic arthritis patients suggested that both have phenotypically similar clonally expanded T cells, also suggesting that AS has an autoimmune signature driven by self-reactive T cells [36]. In addition, the strategy of combining scRNA-seq with CITE-seq has been heavily used to decipher the differences in immune molecules and signaling pathways between plaque and blood tissues [47, 51, 52]. The multiomics approach of combined scRNA-seq provides a powerful tool to dissect the immune response of T cells in AS. With the advancement of technology, it is foreseeable that more sequencing technologies will be added to this field of research in the future to more comprehensively reveal the complex immunoregulatory mechanisms of AS.

### Analysis of intraplaque T-cell communication

Cell‒cell communication (CCC) analysis predicts interactions between different cells by assessing the pairing and expression of receptors and ligands in different cell types. Compared with traditional CCC research, analysis-based scRNA-seq comprehensively and finely examines tissues with broader genome coverage and single-cell resolution. And this region of detection is not limited to the plaque itself, but also includes structures such as the arterial epicardium, ATLO and PVAT.

Analysis of T cell communication networks targeting plaques has led to a clearer picture of the intrinsic immune mechanisms underlying plaque progression. In carotid atherosclerotic plaques, myeloid cells may attract CCR5^+^T cells through the expression of CCL3 and trigger reciprocal activation through specific interactions with T cells (SIRPA-CD47 and ICAM1-ITGAL), resulting in the generation of toxic T cells [48] (Table 5). The cellular interactions between ASYM plaques and SYM plaques differ [47]. In ASYM plaques, T-cell interactions driven by TNF and IFN-γ ligands may promote a proinflammatory phenotype. The expression of IL-1B on macrophages may enhance T-cell effector function through binding to IL-1RAP. In SYM plaques, the PSEN1–Notch interaction within T cells contributes to T-cell activation, differentiation, and PD-1 expression. Moreover, T cells may activate macrophages through VCAN-TLR interactions to promote plaque rupture. Differences in these interactions create different plaque T-cell environments. The specific binding of LGALS9–CD44 to Treg cell subsets enhances their function and stability [52]. Furthermore, differences in the results of cellular communication analyses in scRNA-seq studies at different AS stages may be due in part to differences in the methods and resources used to make communication predictions [62]. The reduced interactions within T cells and with myeloid cells in plaques may be due to the selection of databases that do not contain T-cell–MHC interactions, and the lack of a priori knowledge is also important.

**Table 5:** T-cell communication in atherosclerosis

Gene/Marker	Cell Type/Source	Functional Characteristics	Potential Drug/Application	Research Evidence	Ref.
IL-B-IL-1RAP,PSEN1-Notch,VCAN -TLR	Myeloid-T cell interaction	Enhance the effector function of T cells，promote T cell activation and differentiation, promote plaque rupture	Weaken the activation, differentiation and effector functions of T cells,stabilize the plaque	scRNA-seq of carotid atherosclerotic plaques，CCC analysis	[47]
CCL-3-CCR5,SIRPA-CD47,ICAM1- ITGAL	Myeloid-T cell interaction	T cell localization,Triggers reciprocal activation; generates toxic T cells	Target for reducing toxic T cell generation	scRNA-seq of coronary plaques，CCC analysis	[48]
LGALS9–CD44	Treg interaction	Enhances Treg function and stability	Target for modulating Treg activity	scRNA-seq of CAD patient PBMC, CCC analysis	[52]
SELL-SELPG interaction	CD4+T cell-myeloid interaction	Sex-specific (Male); may explain sex difference in AS progression	Sex-specific intervention target	scRNA-seq of CAD patient PBMC, CCC analysis	[52]
CCL5–CCR1/CCR5	T cell-macrophage interaction	Chemokine signaling in plaque core; sustains inflammation	Target for reducing immune cell aggregation	Spatial and scRNA-seq in human plaques	[63]
CCL5–ACKR1,CXCL12–CXCR4	Adventitial T cell recruitment	Mediates T cell recruitment and retention in adventitia	Target for modulating T cell localization	Spatial and scRNA-seq in human plaques	[63]
CCL2–CCR2,CXCL10–CXCR3,ICAM-1 - LFA-1	Myeloid-T cell interaction	Chemotactic recruitment of Th17/Tfh cells to inflammatory sites,adhesion of T cells	Target for inhibiting T cell recruitment	CCC analysis of human plaques	[64]

AS, atherosclerosis; CAD, coronary artery disease; CCC, cell‒cell communication; PBMC, peripheral blood mononuclear cells; scRNA-seq, single-cell RNA sequencing; Tfh, follicular helper T cell; Th, helper T cell

The cellular communication network may exhibit distinct spatial distributions and functional characteristics in the arterial adventitia and plaque regions. By integrating single-cell and spatial transcriptomic data, Bleckwehl et al. [63] revealed a significant enrichment of chemokine systems such as CCL5–ACKR1 and CXCL12–CXCR4 in the adventitial area, which may mediate T-cell recruitment and retention. In contrast, within the plaque core, notable activation of the CCL5–CCR1/CCR5 chemokine signaling axis was observed between T cells and macrophages—particularly SPP1+ and foam-like macrophages—suggesting its involvement in sustaining inflammation and promoting immune cell aggregation. Spatial co-localization analysis further demonstrated interactions between T cells and M4 macrophages in both plaque and adventitial regions, with more pronounced associations in advanced lesions. Thus, region-specific chemokine-receptor axes coordinately regulate T-cell recruitment and function in a spatially dependent manner, collectively driving the progression of atherosclerosis.

The response of ATLOs to inflammatory signals within the vascular microenvironment likely contributes to the progression of AS. Investigating the communication networks within ATLO structures is crucial for elucidating the relationship between them. Sun et al. [64] integrated 28 scRNA-seq datasets from five vascular disease models, including AS, and identified the consistent presence of ATLOs in the arterial adventitia across all models. During vascular remodeling, T cells, acting as the functional core of ATLO structures, form extensive communication networks with B cells, myeloid cells, and stromal cells through various ligand-receptor mechanisms. First, the interaction between T cells and B cells is key to the germinal center reaction. Follicular helper T cells (Tfh) interact with germinal center B cells through signaling axes such as CD40L-CD40, IL-21-IL-21 R, and CXCL13–CXCR5, promoting B cell maturation. Second, the interaction between T cells and myeloid cells is mainly characterized by chemotaxis and activation. Myeloid cells use CCL2–CCR2 and CXCL10–CXCR3 to chemotactically recruit Th17 and Tfh to inflammatory sites; subsequently, dendritic cells complete the initial activation of T cells through the MHC-II–TCR and CD80/86–CD28 signaling axes. Finally, the interaction between T cells and stromal cells provides spatial positioning. Neo-endothelial cells express CCL21–CCR7 and CXCL12–CXCR4 under mild LTβR stimulation, guiding T cells through high endothelial venules; adhesion signals mediated by ICAM-1–LFA-1 further anchor them in adventitial follicles, ensuring the precise localization of ATLOs around atherosclerotic plaques. In summary, the T cell-centric communication network orchestrates the formation of adventitial ATLOs, facilitating persistent local immune surveillance.

During the progression of AS, cell communication within PVAT also undergoes significant alterations. Through scRNA-seq analysis of human coronary artery PVAT, Fu et al. [65] observed attenuated interactions between T cells and other cell types—particularly fibroadipogenic progenitor (FAP) cells—in atherosclerotic conditions. Conversely, SPP1+ macrophages promote the migration and proliferation of FAP cells via OPN–CD44/integrin interactions, thereby exacerbating PVAT fibrosis and coronary artery stenosis. However, the communication network within PVAT under atherosclerotic conditions remains insufficiently explored and warrants further investigation.

The integration of different resource databases and consistent analysis methods may be a trend for future CCC analyses [62, 66]. Through the integration of multi-omics datasets, particularly those incorporating spatiotemporal dimensions, investigators are poised to holistically unravel the sophisticated symphony of cellular interactions within arterial subsets and their intricate crosstalk with the adjacent milieu [67].

## T-cell subsets in the peripheral blood of human with AS

Peripheral blood mononuclear cells (PBMC) offer insights into disease and treatment without the need for tissue dissociation, preserving cell subset transcriptional information. While CyTOF has been used to characterize AS immune profiles in blood, it lacks the depth to reveal the transcriptional details of cell subsets [68]. An scRNA-seq study of circulating T cells in patients with AS revealed that CD4^+^T cells predominated [47], and that the proportion of total expanded T cells (23% vs. 29%) was lower than that in plaques [36]. In addition, some T-cell subsets, such as Th17-like and MAIT cells, are enriched specifically in the blood, and these subsets are identified mainly in the blood [36]. Plaque T cells display distinct activation states and overlap with exhaustion, whereas in the blood they exhibit autoinhibitory and quiescent states [36, 47]. Furthermore, Vallejo et al. [51] identified 27 T-cell subsets in PBMC samples from coronary artery disease (CAD) patients receiving lipid-lowering therapies (LLT), and CXCR3^+^CD8^+^T cell abundance was reduced after LLT. Specific gene expression differs in response to LLT. For example, the inflammatory cytokine IL32 was significantly increased. JUNB and LCK, which promote Th17 cell development and are involved in T-cell activation, were upregulated in CAD patients but decreased in LLT patients. However, the expression of RUNX is reduced in disease and increased during treatment [69]. These findings suggest that CAD-specific gene expression modulates the augmentation of proinflammatory T-cell effector functions in the peripheral circulation, and that this enhancement is attenuated following LLT. Exploring the immune environment of PBMCs will help in the discovery of key cell subpopulations and transcriptional markers in PBMCs as biomarkers for clinical diagnosis and treatment to better guide patient treatment and risk assessment.

## Sex differences in T-cell subsets

Sex bias has been well explored in the field of cardiovascular disease, including in factors such as smoking, hypertension, hyperlipidemia, the gut microbiota, and the vascular inflammatory response, and the close relationship between estrogen levels and AS has emerged as an important factor contributing to sex bias [70, 71]. Estrogen receptors are widely expressed in different T-cell subsets, and their T-cell-mediated immune regulation affects AS progression [72]. However, the mechanisms by which sex or estrogen bias affects AS progression through T-cell gene expression regulatory pathways have not been fully explored. Saigusa et al. applied scRNA-seq to analyze circulating CD4^+^T cells in statin-treated patients (including those with CAD), suggesting a correlation between sex and the abundance of specific CD4^+^T cell subsets [52]. Among females, the frequencies of the CCR2^+^Tem, MMP9^+^T, and PDL1^+^Tem clusters were reduced. DEG analysis revealed driver genes in CAD patients of different sexes. TCF7, LTB, and GNAI2 expression was upregulated in male CAD patients, whereas IFITM2, IFITM3, and SORL1 expression was significantly upregulated in female CAD patients. The difference in TCF7 expression between different sexes is most significant, and TCF7 may affect AS progression by driving the development of T-cell lineages and participating in the functional maintenance of Tregs [73]. In addition, CCC analysis revealed a ligand‒receptor interaction (SELL‒SELPG) between some CD4^+^T cell clusters and myeloid clusters in CAD patients that was stronger in males than in females, which may partly explain the sex difference in AS progression [52]. In other scRNA-seq studies of AS, sex-based differences in the EC and SMC phenotypes have been reported, and these results have improved our understanding of sex bias in AS disease [74, 75]. However, these results still need to be confirmed by further studies. An increasing number of studies have explored the impact of sex bias in the immune system on cardiovascular disease, providing a direction for further individualized precision immunotherapy.

## Exploration of single-cell sequencing for AS therapy

Current pharmacologic treatments for AS are focused on combating inflammation. The development of precisely targeted immunotherapies that act exclusively at the lesion site and/or the patient’s bloodstream to reduce systemic responses is a potential therapeutic strategy. ScRNA-seq studies have led to a new understanding of the mechanisms of inflammation in AS in mice and humans. New immune cell subpopulations and key disease markers have been identified by sequencing analyses of immune cells from patients with different clinical states, providing directions for therapies that target immune cells.

### Immunotherapy strategies targeting key cell subsets

The key mode of action of traditional AS vaccines is to inhibit Th1 proinflammatory immune responses and enhance antigen-specific Tregs [76]. Targeting the inhibition of cell populations that promote AS progression and are associated with a poor prognosis can provide an entry point for immunotherapy. Xiong et al. [53] identified 2 key proinflammatory T-cell clusters in 3 scRNA-seq datasets. The IFI44L^+^CD8^+^T cell subset with upregulated IFN signaling pathway increases with the progression of AS, indicating its pro-AS characteristics. In CD4^+^T cell clusters, differentiation of SELL^+^CD4^+^T cells with high differentiation potential toward terminal GZMA^+^CD4^+^T cells was accompanied by the enrichment of inflammatory signals and AS-associated pathways, suggesting that proinflammatory GZMA^+^CD4^+^T cells may promote AS progression. Tan et al. [50] identified 3 subsets of plaque-specific T cells (CD8^+^GZMK^+^NR4A1^+^T, CD8^+^GZMK^+^MALAT1^+^T, CD4^+^CDKN1A^+^NR4A1^+^T) in carotid atherosclerotic plaque tissue, which are in the final stages of differentiation and are enriched in T-cell activation, differentiation, depletion, antigen recognition, proinflammatory, and effector pathways, suggesting that they may constitute a key pro-AS population. By targeting such pathogenic T cells, it may be possible to control cardiovascular disease by inhibiting the progression of inflammation. Notably, T-cell subsets with exhaustion phenotypes have been identified in most atherosclerotic plaque studies and are preferentially distributed in the cores of AS plaques [47, 50, 62]. The widespread use of PD-1 inhibitors in tumors has been shown to increase vessel wall inflammation and promote plaque rupture, possibly because intervention in exhausted T-cell subsets leads to their activation, thus exacerbating AS [77]. However, in a recent study, treatment of tumor patients combined with carotid atherosclerotic plaques using FcγR combined with an anti-PD-1 monoclonal antibody (mAb) reduced the size of AS plaques, possibly because CD64 on the surface of myeloid cells in close proximity to PD1^+^T cells within the plaque captures the anti-human PD-1 IgG4 mAb as a surrogate ligand for PD-1, which triggers the inhibitory function of PD-1 and ultimately leads to decreased secretion of inflammatory cytokines and plaque regression [37]. Therefore, anti-PD-1 mAbs with FcγR-binding capability (such as Nivolumab) may promot plaque regression, whereas those without FcγR-binding capability (such as Tislelizumab) showed no such effect [37]. Different classes of immune checkpoint inhibitors modulate atherosclerosis progression through distinct mechanisms of T cell subset intervention. For instance, CTLA-4 inhibitors (e.g., ipilimumab) may promote atherosclerotic progression by impairing Treg cell-mediated suppression and enhancing effector T cell activation. This mechanism provides a plausible explanation for the increased incidence of cardiovascular events observed in previous studies employing combination therapy with CTLA-4 and PD-1 inhibitors [77]. Beyond the strategies discussed above, targeting rare but specialized T-cell subsets within plaques—such as NKT cells and γδ T cells—or modulating T-cell functions through alternative co-stimulatory or co-inhibitory immune checkpoints (e.g., CD40–CD40L, CD27–CD70, CD30–CD30L, OX40–OX40L, TIM-1, BTLA, and Tigit) holds significant promise for the development of novel immunotherapeutic strategies [78]. Overall, the role of T-cell subsets in AS is often extensive and unpredictable, and more research is needed to clarify the characteristics of T-cell subsets.

### Key genes as diagnostic biomarkers and potential targets for treatment

Bioinformatics technology has identified key genes of immune cells and immune regulatory factors, which can be used to predict or screen for markers for effective disease prognostication or diagnosis, providing ideas for new diagnostic models and targeted immunotherapy strategies [79]. Wang et al. [45] reported DEGs in immune cell populations between stable and unstable carotid AS patients in public databases, and revealed correlations between certain core genes and immune cells. For example, CD8^+^T cells were positively correlated with IGFBP6 and CNN1 and negatively correlated with SLC3A2. Advanced miRNAs were filtered out by constructing a miRNA-core gene network, through which effective candidate genes were obtained. Key DEGs (CD68, PAM and IGFBP6) in unstable plaques were validated as effective diagnostic markers. Finally, gene‒drug interactions were utilized to mine therapeutic agents. MDM2, KCNA5 and ANO1 correspond to abundant targeted drugs and could be used as potential therapeutic targets. A similar approach confirmed the anti-AS effect of salvianolic acid B, which targets MMP9 and MMP12 [80]. In a post carotid endarterectomy patient, Ma et al. [81] analyzed CITE-seq data from SYM plaques and reported that plaques highly expressed the toxicity-related gene CXL1. Concurrent scRNA-seq studies in the aortas of HFD-fed LDLR^-/-^ and ApoE^-/-^ mice revealed that T cells were enriched with proliferation-related genes (TCF7, Rag1, and Sox4) and CCR9. The roles of TCF7 and XCL1 in AS have not been fully studied. Weighted gene co-expression network analysis suggested that these genes are regulated by the AKT and NF-κB pathways respectively. These two pathways are activated after stimulating T cells with LPS in vitro, indicating that TCF7 and XCL1 may mediate the progression of AS and serve as potential therapeutic targets [81]. The signature genes of T-cell subsets in plaques were analyzed by the Bike database, and the highly expressed signature gene IRF1 was positively correlated with a high risk of cerebrovascular events, whereas the weakly expressed ETS1 was negatively correlated [50]. The signature gene CD42 in SYM plaques and the downregulated gene CD2 were negatively associated with a high risk of cerebrovascular events [50]. All of these key genes are potential targets for future immunotherapy. However, both validated biomarkers and potential therapeutic targets require further experimental and external validation, which will be the direction of future research.

## Challenges of scRNA-seq in AS

In recent years, with the rapid development of single-cell sequencing technology, this technology has become an important tool in disease research. However, compared with studies in fields such as tumors and infections, single-cell studies on T-cell populations in atherosclerosis are still in their infancy. Although some progress has been made in the classification, function, recruitment and cell fate of T cells in the study of atherosclerosis at the tissue level, a comprehensive and in-depth elucidation of the mechanism of action and fate lineage of T cells is still insufficient. Breakthroughs in this field are expected through single-cell sequencing studies based on experimental animal and human samples, or through in-depth mining of existing datasets [14].

However, many challenges remain in practical research. First, the heterogeneity of tissue samples across different species and batch preparations, coupled with the notable spatiotemporal heterogeneity of T cells within arteries, leads to disparities in T-cell microenvironments, thereby compromising the comparability of results across species and studies. Current human arterial specimens are largely procured at end-stage disease via endarterectomy or autopsy, thereby over-representing plaques with large necrotic cores and symptomatic phenotypes. Early, asymptomatic lesions remain virtually inaccessible, restricting the temporal resolution of lineage tracing and trajectory inference. Although emerging spatial transcriptomics preserves in situ spatial information, its transcript-capture sensitivity is still inferior to that of conventional scRNA-seq, leading to the under-detection of low-abundance transcripts. Second, different single-cell suspension preparation strategies may induce cell bias, leading to underestimation and bias of T-cell subsets during sequencing. For instance, enzymatic digestion and mechanical disruption tend to preferentially damage or deplete fragile T-cell populations—such as Tregs—resulting in systematic skewing of subset proportions. Furthermore, the dissociation process artificially induces stress-response transcriptional programs, including upregulation of immediate-early genes (e.g., FOS, JUN), which can be misinterpreted as genuine “T-cell activation” signals. Even if these losses are disregarded, rare populations including γδ T, NKT and MAIT cells frequently fall below the detection threshold, resulting in insufficient statistical power and false-negative conclusions. In addition, the processing and analysis of single-cell sequencing data require highly specialized skills. Although cross-platform large-scale data integration and diversity analysis tools provide flexibility, they also increase the incomparability of analysis results. For example, the interpretation of results for T-cell subsets may vary due to the lack of uniform standards for data analysis and cell annotation. Finally, scRNA-seq itself also has limitations. It cannot fully reflect the actual functional status of T cells at the protein level and lacks tissue spatiotemporal information, which hinders the study of T-cell interactions and dynamic processes.

## Future of scRNA-seq in AS

With the development of sequencing technology and bioinformatics, the application of scRNA-seq has advanced rapidly. First, the cost of single-cell sequencing has gradually decreased. Second, data processing procedures such as removing multiples and dead cells, eliminating batch effects, and addressing missing transcriptional data will become more standardized, and data storage will be unified. More importantly, with the construction of online data analysis platforms and the optimization of analysis methods, the professional threshold required for single-cell sequencing data analysis will be gradually reduced. On the basis of these advances, scRNA-seq will be more widely used, and the era of big data analysis of public datasets is approaching, which will provide strong support for novel clinical diagnostic strategies, mining of potential therapeutic targets, and drug development in atherosclerosis [82, 83].

Single-omics studies usually have their own limitations, and co-sequencing via multi-omics is becoming a trend. Proteomics studies have relied mainly on antibody-based strategies, microfluidic platforms, and mass spectrometry to measure proteins. However, their small volume and large bias limit our ability to understand how gene expression translates into cellular phenotypes. Recent spatial transcriptomics techniques spatially annotate gene expression at near-single-cell resolution but do not reach the depth and whole-transcriptome coverage of scRNA-seq [84]. Combining scRNA-seq with proteomics, spatial transcriptomics, and other methods allows these approaches to complement each other to more fully reveal the mechanisms by which different cellular subsets synergistically contribute to disease phenotypes [85–88]. Theofilatos et al. [89] relied on a multiomics strategy to mine key protein markers that predict the risk of cardiovascular events in sex-diverse AS patients. Using a similar strategy, key genetic markers in T-cell subsets can also be explored and validated. T cell differentiation, function and memory formation are not only regulated by transcription factors and cytokines, but also depend on the dynamic remodeling of the epigenetic landscape [90]. Epigenetic sequencing, including TAB-seq to obtain information on DNA methylation, CHIP-Seq to obtain information on histone modifications, and noncoding RNA sequencing, can also be integrated into multi-omics frameworks to delineate the spatiotemporal epigenetic heterogeneity of T-cell subsets across disease stages, thereby identifying actionable epigenetic targets and yielding novel biomarkers for atherosclerosis immunotherapy [91]. Recent breakthroughs in single-cell epigenomic technologies, such as scATAC-seq, have enabled the resolution of chromatin accessibility dynamics in T cell subsets at single-cell resolution, thereby revealing the activation states of disease-associated regulatory elements [92]. Simultaneously, transcriptomic analysis of epigenetic enzymes via scRNA-seq has provided new insights into disease mechanisms. For instance, Bonfiglio et al. [93] identified a distinct T cell subset characterized by high expression of the histone methyltransferase EZH2 through scRNA-seq analysis of human atherosclerotic plaques. Subsequent knockout of Ezh2 in CD4^+^ T cells in ApoE^−^ /^−^ mice alleviated epigenetic repression of Zbtb16 (encoding PLZF) and Il4 genes, promoting iNKT2 and Th2 cell differentiation, inducing a type 2 immune response and anti-inflammatory macrophage polarization, and ultimately attenuating atherosclerosis progression. Integrating single-cell multi-omics (e.g., scRNA-seq combined with scATAC-seq) with targeted epigenetic modulation strategies may enable future “epigenetic reprogramming” to restore T cell homeostasis, offering a novel paradigm for precision immunotherapy in AS. With the development and application of multiomics sequencing technology, future research in the field of atherosclerotic diseases will reveal a more diverse landscape, aiding the future of immunization against atherosclerosis [94, 95].

With the rapid development of machine learning algorithms and artificial intelligence (AI), there is a new opportunity for big data analysis as well as the development of disease models. Large-scale training models developed on the basis of multimodal and multiomics data can be applied to single-cell downstream analysis, covering multiple aspects of multiomics integration, cell type annotation, gene expression enhancement, gene module inference, protein interaction network analysis, prediction of tissue drug response, and single-cell perturbation, such as scFoundation [96], ScGPT [97], and PINNACLE [98] models. In addition, AI-driven single-cell research is expected to drive the development of personalized treatments for diseases. For example, in cancer research, Tang et al. [99] constructed a comboSC framework based on graph combinatorial optimization algorithms to optimize combination therapies for patients with low to moderate immune scores by analyzing scRNA-seq data to infer the relationships between drug candidates and heterogeneous patient populations. Recently, Wang et al. [100] constructed an immune age prediction model (siAge) for identifying immune dysfunction on the basis of multiomics data from 220 healthy individuals, providing a new direction for immune health assessment and individualized immune intervention. It is foreseeable that AI-driven single-cell research will change the existing research paradigm in the future and may have a significant impact on current cardiovascular research and clinical translation.

To address the critical need for causal validation of scRNA-seq findings, future studies should integrate descriptive single-cell analyses with functional in vivo approaches. Genetic lineage tracing, adoptive transfer experiments, and targeted depletion of specific T-cell subsets in animal models remain essential for establishing the pathogenic or protective roles of disease-associated T-cell populations identified through scRNA-seq. Furthermore, the application of emerging technologies—including CRISPR-based gene editing, single-cell perturbation assays, and single-cell virtual knockout in preclinical models—will be instrumental in translating descriptive observations into mechanistic insights and therapeutic applications.

## Summary

T-cell subsets, as important components of the immune microenvironment of AS, influence the development of this disease. High-throughput single-cell sequencing technologies have revealed the heterogeneity and functional characterization of atherosclerotic plaque T-cell subsets and provided new directions for the exploration of future disease treatments. However, the current major sequencing efforts are focused mainly on transcriptomic aspects, which are not yet able to define deeper T-cell subsets. With the development of multiomic technologies, joint sequencing techniques combining genomic, proteomic, metabolomic, epigenetic, and spatial transcriptomic data will more comprehensively reveal new T-cell subsets and cellular functional differences, and will also be able to more reliably elucidate issues such as immune-antigenic specificity, the mechanisms of disease progression across different clinical states, and sex differences in disease. In the future, single-cell sequencing technology will be more widely used in the field of immune cells in AS. This will not only facilitate the development of novel targeted immunotherapies but also accelerate the identification of key molecular targets for drug intervention and the discovery of diagnostic biomarkers. By bridging these gaps, the integration of single-cell technologies promises to underpin a new era of precision medicine, ultimately expanding the therapeutic and prognostic landscape for a broad spectrum of AS patients.

## Data Availability

No additional data is available.

## Declarations

### Use of large language models, AI and machine learning tools

None declared.
